# Environmental toxicology: how pervasive organic environmental pollutants cause toxicity at the molecular, cellular and organism level

**DOI:** 10.1002/2211-5463.13883

**Published:** 2024-09-06

**Authors:** Francesco Michelangeli

**Affiliations:** ^1^ Chester Medical School University of Chester UK

## Abstract

This special issue in *FEBS Open Bio* was conceived to highlight some of the current research and future directions regarding research in the field of environmental toxicology of some organic pollutants, in relation to human health and disease. It has long been established that man has been exposed to many new (un‐natural) organic chemicals since the beginning of the Industrial Revolution, many of which are found in a vast and diverse range of products, such as agrochemicals, pharmaceuticals, plastics and electronic components, which have been instrumental in driving man's exponential advances in technology over the last 150 years. However, an unforeseen consequence of these advances is that our ecosystem has been exposed to a vast number of these organic chemicals, many of which appear to be persistent within the environment, as well as bioaccumulating in living organisms, including man.

**Abbreviations**
BFRs: brominated flame retardants
EDCs: endocrine‐disrupting chemicals
MDCs: metabolic disrupting chemicals
SERCA: sarcoplasmic/endoplasmic reticulum Ca^2+^ ATPase

Over the last 40 years, it has become evident that some of synthetic organic chemicals that ultimately permeate the environment may cause detrimental effects on living organisms and lead to health issues. Some of the early research findings with pesticides and plastics manufacturing chemicals suggested that some of these chemicals may cause health issues due to their ability to disrupt the endocrine system [endocrine‐disrupting chemicals or (EDCs)] possibly by mimicking the effects of hormones such as Oestrogen (i.e. xenoestrogens). These effects have led to the suggestion that some of these chemicals could cause problems with male fertility and may be a factor in altering normal developmental processes, as well as possible implications in the cause of some forms of cancer.

More recently, it has been proposed that some of these chemicals can also act as EDCs through disrupting other aspects of endocrine signalling processes such as Ca^+^ signalling. In the first of these reviews by Michelangeli et al. [1] the focus is on how some alkylphenols and brominated flame retardants (BFRs) can cause cell death to a variety of cell types, especially when acute exposure occurs, through activation of cell death pathways such as apoptosis and autophagy. In addition, it has also been shown that the endoplasmic reticulum (ER)‐stress response also plays a role. From a wide range of research studies, it was identified that all these processes were due to the release of Ca^2+^ from the ER leading to abnormally high cytosolic levels of [Ca^2+^], activating Ca^2+^‐dependent cell death processes. The major protein identified to illicit this rise in cytosolic Ca^2+^ was the sarcoplasmic/endoplasmic reticulum Ca^2+^ ATPase (SERCA). As a personal endeavour, my group was responsible in trying to understand how some of these EDCs interact with the Ca^2+^ATPase at a molecular level to inhibit this pump and lead to the activation of specific cell death pathways.

In the second review article by Erradhouani et al. [2] the finding that many of the same organic pollutant chemicals as EDCs are also now described as metabolic‐disrupting chemicals (MDCs) due to their effect in exasperating diseases commonly referred to as metabolic syndrome, that is type 2 diabetes mellitus, cardiovascular disease and fatty liver disease, etc. The review focusses on the effects of these environmental chemicals on organs and tissues leading to these metabolic disorders as well as on, the as yet little studied, affect of these chemicals in the intestines. The key questions that need addressing are; how can these chemicals be metabolised in the intestines, as well as how these chemicals might be absorbed from the intestines into the body to affect key organs and tissues. A further emphasis of this review is to identify a potential model system to further study how the intestines deal with and metabolise the MDCs and proposes that the zebrafish would make a good system given its similarities in the physiology and metabolism occurring in human intestines.

Although there is a great body of research in investigating and understanding the molecular mechanisms by which individual environment polluting chemicals cause adverse effects on cells and organisms, in the real world, we are not just exposed to one of these chemicals at any particular time, but rather we are being continually exposed to a cocktail of chemicals at differing concentrations. Relatively, little research has been undertaken to investigate what the effects chemical mixtures might have on biological and biochemical processes within humans. In the third review of this issue, Eze and Vinken [3] present information regarding the toxicity of e‐waste, which comprises a range of organic and inorganic pollutants, and present some effect‐based monitoring strategies to assess the effects of complex chemical mixtures on living organisms.

## Conflict of interest

The authors declare no conflict of interest.

## Author contributions

FM wrote the article.
